# Goserelin Ovarian Ablation Failure in Premenopausal Women With Breast Cancer

**DOI:** 10.7759/cureus.19608

**Published:** 2021-11-15

**Authors:** Aanchal Gupta, Sindhura Bandaru, Sukesh Manthri

**Affiliations:** 1 Internal Medicine, Faculty of Medicine, St. Martinus University, Willemstad, CUW; 2 Internal Medicine, Southern Illinois University School of Medicine, Springfield, USA; 3 Oncology, Mary Bird Perkins Cancer Center, Houma, USA

**Keywords:** gonadotropin releasing-hormone (gnrh) agonist, new breast cancer treatment, triptorelin, hormone receptor-positive breast cancer, failure of ovarian suppression, ovarian suppression, breast cancer research, breast cancer, gnrh analogues, goserelin

## Abstract

Breast cancer is the most prevalent cancer known worldwide in women. It is a heterogeneous, phenotypically diverse disease composed of several biologic subtypes that have distinct behavior and response to therapy. Hormone receptor-positive (i.e., estrogen [ER] and/or progesterone [PR] receptor-positive) breast cancers comprise the most common types of breast cancer, accounting for 75% of all cases. This makes endocrine therapy the standardized treatment for patients with ER+/PR+ breast cancer. Drugs that block estrogen receptors or that lower estrogen levels are the mainstay of treatment. High-risk patients benefit from the addition of ovarian function suppression (OFS)/ablation to either an aromatase inhibitor (AI) or tamoxifen. This case report discusses a 36-year-old premenopausal female who presented with an abnormal right breast lump in the upper outer quadrant of the right breast. Due to high suspicion of malignancy, a biopsy was performed which showed features of both lobular and ductal carcinoma with ER and PR positivity, HER 2 was negative. The patient underwent mastectomy with axillary lymph node removal due to concern for multifocal disease. No clinically relevant genetic mutations were present. Oncotype DX breast recurrence score was 16 and no chemotherapy was offered. Due to large tumor size, young age OFS with goserelin 3.6mg/28 days and letrozole 2.5 mg once daily was recommended. After 16 months of treatment, the patient developed a failure of goserelin-induced ovarian suppression. This case report highlights the possibility of the development of hormonal resistance after long-term use of goserelin.

## Introduction

Breast cancer has been established as the most common type of cancer present in women in the United States (US) and worldwide. It is estimated that one in eight US women will develop invasive breast cancer in their lifetime [1]. There are a variety of risk factors associated with the development of breast cancer, gender, and aging is the most common ones. An inherited mutation in BRCA1 and BRCA 2 is the most common cause of the development of hereditary breast cancer. There are four main molecular subtypes of breast tumors: HER (human epidermal growth factor receptor 2), basal cell type, luminal A and B types. Luminal A and B types are hormone receptor-positive breast cancer (ER/PR positive) and make up for the majority (75%) of breast tumors [2]. Therefore, this makes hormonal therapy a prime therapeutic target for most breast cancer treatments. Endocrine therapy combining aromatase inhibitor (AI) or tamoxifen with gonadotrophin-releasing hormone agonist (GnRHa) has become the mainstay therapy in premenopausal women for ovarian suppression [3]. For AI to work and be therapeutic in premenopausal women, ovarian estrogen production must be suppressed, which is attained by using GnRHa. In a nationwide Austrian trial, the study demonstrated the use of endocrine therapy proved to be more effective than the standard chemotherapy and improves the prognosis in younger women with breast cancer [4]. Suppression of ovarian function trial (SOFT) and tamoxifen and exemestane trial (TEXT) adjuvant trials showed that ovarian function suppression improves disease-free and overall survival [5].

There have been very few cases reported regarding the failure of ovarian suppression with a GnRHa and AI in a premenopausal woman. This case report highlights the possibilities for failure such as age, autoantibodies to the injection site, BMI and route of administration that could attribute to the development of incomplete ovarian suppression.

## Case presentation

**Figure 1:**
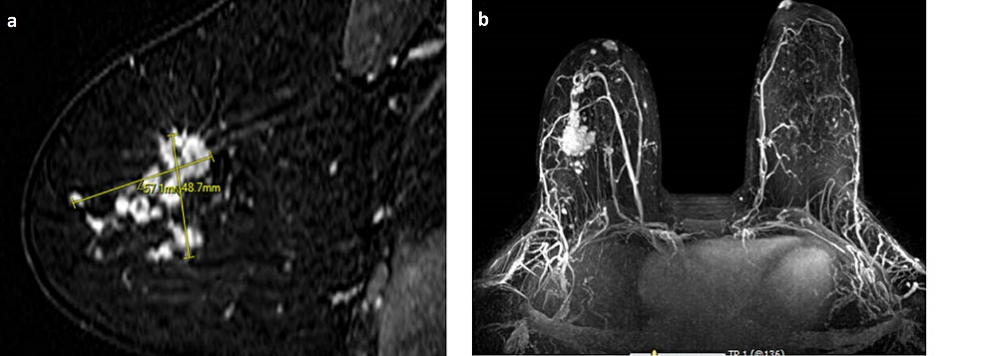
(a) Magnetic resonance imaging of the right breast with heterogeneous mass with irregular margins measuring 57.1 mm x 48.7 mm. (b) Magnetic resonance imagining with contrast in axial view.

A 36-year-old G4P2 premenopausal woman with a family history of colorectal, hepatobiliary cancers felt an abnormal right breast lump. Diagnostic mammogram and ultrasound showed a hypoechoic lesion in the upper outer quadrant of right breast measuring 14 mm x 13 mm x 18 mm and 5 x 4 mm satellite lesion is noted 6 mm inferior to the dominant mass, BI-RADS 5 highly suggestive of malignancy. Due to concern for multifocal disease, MRI breast with without contrast was done and it showed 2.3 x 1.1 x 2.7 cm irregular-shaped, heterogeneous mass with irregular margins in the upper outer quadrant of right breast, 7 cm from the nipple, 1.2 cm from the skin and there was an additional mass measuring 8 mm x 4 mm x 1.6 cm at 12:00 along with 4 mm lesion, 7 mm from the nipple at 10:00 (Figures 1 (a) and (b)). Right breast biopsy from the dominant lesion showed invasive mammary carcinoma with features of both lobular and ductal carcinoma, Nottingham histological grade 2, estrogen receptor 90%, progesterone receptor 100%, HER2 2+ by IHC but negative by FISH, Ki-67 50%.

Status post right breast simple mastectomy and axillary lymph node evaluation. Surgical pathology showed a multifocal invasive mammary carcinoma of the breast with ductal and lobular features, size of largest invasive carcinoma was 55 mm, size of additional invasive foci was 1.5 mm, Nottingham histological grade 2 of 3, low to intermediate nuclear grade DCIS without central necrosis measuring at least 6 mm, margins uninvolved, one benign sentinel lymph node. Pathological staging (m)pT3 (sn)N0. Oncotype DX breast recurrence score of 16 (for patients <50 years of age, benefit from chemotherapy 1.6%). Genetic testing did not reveal any clinically significant mutations. The patient has received adjuvant PMRT 5000 cGy dose, 25 fractions along with 1000 cGy scar boost. Based on TEXT/SOFT data ovarian suppression could be considered in patients with high-risk features. The patient's tumor was multifocal, and the largest lesion measured 5.5 cm. The patient elected against surgical menopause. The patient was started on ovarian suppression with goserelin 3.6 mg every 28 days along with letrozole 2.5 mg once daily.

Sixteen months after initiation of ovarian suppression patient started having a regular menstrual cycle while on goserelin. No medication interaction or missed doses were noted. The patient's BMI was 41.1 and BSA was 2.19 m^2^. Estradiol was 92 pg/mL and follicle-stimulating hormone (FSH) was 4.3 mIU/mL in the premenopausal range. Few prior case reports showing failure of goserelin ovarian ablation in a premenopausal woman were reported. Given high-risk disease, discussed benefit of continued ovarian suppression plus AIs versus tamoxifen alone, and discussed the role of oophorectomy which results are reliable and prompt reduction in circulating estrogens. The patient was hesitant and wished to wait before the oophorectomy. She was started on triptorelin 3.75 mg IM once every 28 days.

## Discussion

**Figure 2:**
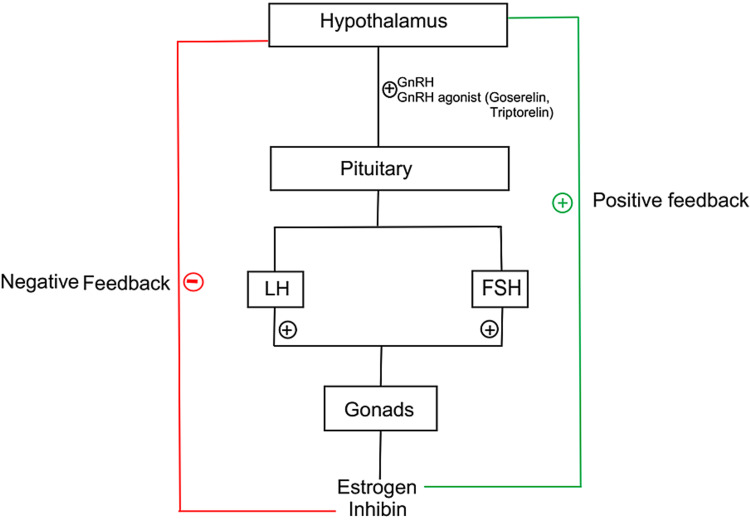
Hypothalamic-pituitary-gonadal axis Gonadotrophin-releasing hormone (GnRH) agonist (GnRHa) works by stimulating the GnRH in the hypothalamus, making bouts of GnRH which in turn stimulate the anterior pituitary to secrete luteinizing hormone (LH) and follicle-stimulating hormone (FSH). LH and FSH act on the ovaries to produce estrogen. The GnRHa tend to stay attached to the GnRH receptors for a longer duration which causes a transient increase in estrogen for a few weeks, which could worsen the hormonal responsive breast cancer transiently. After continuous stimulation of GnRH receptors on the anterior pituitary, they become desensitized to GnRH hormone, shutting down its production of LH and FSH which in turn decreases the production of estrogen causing ovarian suppression and the patient enters chemical menopause.

Some patients who are diagnosed with hormone receptor-positive cancer require adequate suppression of ovarian function, as indicated by low Estradiol (E2) levels, achieved by the use of GnRH agonists (Figure 2), especially young premenopausal women who are at increased risk of adverse outcomes [6]. Other options besides chemical ovarian suppression are surgical oophorectomy and radiation-induced ovarian ablation which cause irreversible menopause, making it a less suitable option for relatively young premenopausal patients. Selective estrogen receptor modulators (SERM) have been used for the longest time in premenopausal women for the treatment of estrogen-positive breast cancer. In 2003, SOFT and TEXT studies were done to determine the superiority of adding ovarian suppression to tamoxifen as well as to establish the role of AIs plus ovarian suppression [7,8]. It was found that after a median of 5.6 years, use of tamoxifen alone or tamoxifen plus ovarian suppression did not affect the disease-free survival except in premenopausal women with higher risks of recurrence [7], but in the updated SOFT analysis, which is an updated analysis with a median of eight years showed a significant difference with higher rates of disease-free and overall survival [8]. It was seen that exemestane plus ovarian suppression was superior to the use of tamoxifen and ovarian suppression with a difference of 7.0 percentage points when tamoxifen is used alone and 2.1 percentage points in comparison to tamoxifen plus ovarian suppression, hence favoring the use of AI over SERM [8].

There have been few cases reported with the failure of ovarian suppression via the use of GnRHa [4,5]. No consolidated literature explaining the causes of failure of ovarian suppression has been found. There are various factors that have been postulated as the probable causes of this failure, including age, BMI, and frequency of GnRHa administration [3]. Goserelin, leuprolide, and triptorelin are the most common GnRHa used for the adjuvant therapy, administered via subcutaneous (SQ) and intramuscular (IM) routes, respectively.

This case report demonstrates a 36-year-old female who attained chemical menopause after the use of Goserelin 3.6mg subcutaneously for 16 months. She suddenly started having normal menstrual cycles and upon testing her FSH and estradiol levels they were in the premenopausal range, raising concern for failed ovarian suppression with resistance against goserelin due to an unknown mechanism. In the SOFT-EST study, it was concluded that out of all the patients, at least 17% of patients demonstrated a greater than the threshold estradiol (E2) level within the five-year period of the trial [4]. Only a few case reports have been documented with failure of goserelin-induced ovarian ablation which resulted in pregnancy or disease metastasis [9,10]. This failure has been hypothesized to be linked to various factors. The average duration of failure that has been mentioned in other case reports was around four months [6]. Age could also be identified as a predictive factor for failure of efficient ovarian suppression (3). Besides age, a high BMI > 25kg/m^2^ has also been associated with decreased efficacy of ovarian suppression [11]. Unfortunately, the case reports in the current literature do not exhibit a clear relationship between BMI and Failure of ovarian suppression with BMI ranging from as low as 18kg/m^2^ to as high as 26kg/m^2^ [3]. We presume that age and BMI may have a synergistic effect on the failure of ovarian suppression. More studies need to be done in order to establish this relationship.

It was found that with the use of GnRHa alone, the LH suppression persists without a recovery but after a few months of treatment, FSH may return to baseline levels which could also stimulate the secretion of estradiol and hence failure of ovarian suppression [12]. This FSH escape has been related to the lack of GnRHa effect on alpha gonadotrophin subunit and FSH beta-subunit gene leading to inadequate suppression [13,14]. The addition of AI to GnRHa also does not suppress the residual ovarian synthesis but rather could lead to increased estradiol production in some cases [12]. Austrian breast and colorectal cancer study group (ABCSG) 12 trial also suggest that BMI has an impact on the efficacy of goserelin plus AI, and it decreases in overweight patients [15].

Patients may also develop resistance to certain GnRHa due to the formation of autoantibodies against the molecular peptide structure of goserelin [16,17]. Our patient was not tested for autoantibodies against goserelin due to financial constraints and limitations of resources. Other GnRH analogs with different molecular structures like triptorelin could be used if the patient continues to deny pursuing any other forms of permanent ovarian ablation and preserve fertility. The use of GnRH antagonists has also been showing promising results and could be considered as an alternative therapy [5]. Moreover, there are no current guidelines that are established for monitoring serum estradiol levels at regular intervals to recognize the failure of OS earlier and avoid any unintended complications [3,5].

## Conclusions

The use of GnRH analogs has been the mainstay therapy for high-risk premenopausal women to achieve temporary chemical ovarian ablation. But treating physicians should perform caution as there is a chance of failure of ovarian suppression with long-term use especially in older premenopausal women and patients with increased BMI. Age and BMI could possibly have a synergistic efffect on failure of ovarian suppression. Further investigation should also be performed to understand the pathophysiology and to explore the possibility of autoantibodies against the GnRH analogs. This report also highlights the need to establish a guideline for estradiol, FSH monitoring at regular intervals to recognize the failure of ovarian suppression early and avoid any unanticipated complications.
